# Relationship Between Sleep Quality and Glycemic Control Among Patients With Type 2 Diabetes

**DOI:** 10.7759/cureus.91282

**Published:** 2025-08-30

**Authors:** Sandhya Sahani, Rida Zahra, Boakye Yiadom Adu, Sadia LNU, Maherullah Kasi, Mahbubur Rahman, Sarah Mehdi, Muhammad Shams ul Haq, Muhammad Ashfaq Subhani, Nimra Kalim, Sher Bano, Eisha Naeem

**Affiliations:** 1 Emergency Medicine, Tomo Riba Institute of Health and Medical Sciences, Naharlagun, IND; 2 Medicine, Zaporizhzhia State Medical University, Zaporizhzhia, UKR; 3 Neurology, Punjab Institute of Neurosciences, Lahore, PAK; 4 Cardiology, Ternopil National Medical University, Ternopil, UKR; 5 Public Health, University of Northampton, Karachi, PAK; 6 General Surgery, Sandeman Provincial Hospital, Quetta, PAK; 7 Cardiac Anaesthesia, National Heart Foundation Hospital and Research Institute, Dhaka, BGD; 8 Family Medicine, Allama Iqbal Medical College, Lahore, PAK; 9 Internal Medicine, Dera Ghazi Khan Medical College, Dera Ghazi Khan, PAK; 10 Internal Medicine, International Medical University Kyrgyzstan, Bishkek, KGZ; 11 College of Medicine, Baqai Medical University, Karachi, PAK; 12 Clinical Psychology, Shifa Tameer-e-Millat University, Islamabad, PAK; 13 Medicine, Shifa International Hospitals, Islamabad, PAK

**Keywords:** cross-sectional, glycemic control, self-management, sleep quality, type 2 diabetes

## Abstract

Background: The quality of sleep has been identified as a significant contributor to the management of type 2 diabetes mellitus (T2DM), particularly in achieving optimal blood glucose levels. Although previous research globally has acknowledged this relationship, there is a lack of adequate studies in low- and middle-income countries, such as Pakistan. The purpose of this study is to investigate the relationship between self-management behavior (as indicated by glycemic control) and sleep quality among individuals with type 2 diabetes mellitus (T2DM).

Methods: This study is a cross-sectional study conducted from January to May 2025 in outpatient healthcare centers in Islamabad, Pakistan, using a convenience sampling methodology. The study included 385 adult respondents with a diagnosis of type 2 diabetes mellitus (T2DM). The structured questionnaire included sections on demographic characteristics, the Pittsburgh Sleep Quality Index (PSQI), and the Diabetes Self-Management Questionnaire (DSMQ). Data analysis was conducted using IBM Corp. Released 2020. IBM SPSS Statistics for Windows, Version 26. Armonk, NY: IBM Corp., employing descriptive statistics, Spearman correlation, Mann-Whitney U tests, Kruskal-Wallis H tests, chi-square tests, and linear regression to investigate the relationship between sleep quality and diabetic self-management practices.

Results: A total of 385 participants took part in the study, comprising 195 males (51%) and 190 females (49%). PSQI and DSMQ were also negatively correlated at a highly significant level (r = -0.119, p = 0.020), and this indicates that poor quality of sleep was interrelated with a low rate of self-management of diabetes. Female respondents also had significantly lower scores in sleep quality and reached higher scores in self-management than male respondents (p < 0.01). There were also significant differences in the PSQI and DSMQ scores, even when marital status was taken into account (p < 0.01). Based on the results of linear regression, it was possible to note that PSQI scores had a significant effect on predicting DSMQ scores (B = -0.452, p = 0.021); thus, the lower the score of the sleep quality measure, the less positive were the diabetes self-care behaviors.

Conclusion: It was found that sleep quality and glycemic control behaviors predict a strong negative interrelationship in T2DM patients in Pakistan. The management of diabetes should consider including a sleep assessment. To achieve improved outcomes, recommended gender- and marital status-sensitive interventions are advised, including a targeted approach to self-management and sleep hygiene.

## Introduction

Type 2 diabetes mellitus (T2DM) is a multifactorial condition that includes insulin insensitivity and alterations in beta-cell dysfunction, causing severe vascular sequelae [1]. The prevalence of T2DM is growing tremendously across the globe, primarily due to obesity levels, urbanization, and sedentary living. The epidemic is spreading to developing nations, with forecasts indicating a significant increase in prevalence worldwide by 2030 [2].

In Pakistan, diabetes is a significant community health threat, with an estimated prevalence of 17.1% in 2019, putting it in the fourth position among world nations. Barriers to effective management can be characterized by a lack of resources, societal challenges, and patient-related issues, demonstrating the necessity to enhance education, telemedicine, and precision medicine practices [3].

Epidemiological data suggest that not only poor quality of sleep but also prolonged or short sleep is linked with an elevated HbA1c level in individuals with type 2 diabetes. This indicates a U-shaped correlation between sleep patterns and glycemic control, which emphasizes the metabolic significance of sufficient and quality sleep [4]. Sleep quality is highly associated with overall health and may be an indicator of underlying medical issues [5].

Glycemic control in the U.S. population with diagnosed diabetes improved considerably between 1999 and 2004, as mean HbA1c levels declined, and a greater proportion met the target glycemic level [6]. While better glycemic control stabilizes early diabetic complications, it is still not entirely understood how they are associated with each other since some people never develop complications despite the glucose levels [7].

The bidirectional relationship between T2DM and sleep disturbances is increasingly being recognized. Sleep deficits contribute to glucose and insulin resistance, which is part of a vicious cycle that further worsens metabolic dysfunction [8]. Studies also confirm that poor sleep quality and perceived 'sleep debt' are linked with elevated HbA1c among patients with T2DM [9]. Similarly, poor sleep quality and low sleep efficiency have been shown in Asian populations to be strongly related to poor glycemic control [10]. Japanese cohorts have also demonstrated worse subjective sleep quality in groups with higher HbA1c levels, independent of confounding factors [11].

Rationale

Sleep quality has recently been recognized as a significant contributor to glycemic control in patients with type 2 diabetes over the past several years. There is an emerging body of evidence that poor sleep is not just a side effect of the complications of diabetes. Still, it may be a substantial contributor to the development of glucose dysregulation. This consequently means that the quality of sleep is currently being incorporated into the larger lifestyle and behavioral change patterns as a means of managing diabetes. Nevertheless, even with such increased awareness, there remains a need to conduct context-specific studies that investigate sleep quality and its influence on glycemic control among different populations. Most existing research has been conducted in the West [12]; unfortunately, there is limited information available about low- and middle-income nations, including Pakistan, where cultural, environmental, and access-to-healthcare factors can significantly influence both sleep patterns and diabetes management.

This research aims to fill this gap by examining the relationship between sleep quality and glycemic outcomes in individuals with type 2 diabetes within a local clinical setting. Exploring this particular population will allow the research to produce evidence that is directly applicable in local health practice and patient needs. The results can promote a more personalized and practical approach to overall diabetes management, with sleep analysis becoming a regular element of a clinical assessment and strategic intervention planning.

Primary objective

To evaluate the correlation between sleep quality and diabetes self-management behaviors (as assessed by the DSMQ), which are essential behavioral correlates of glycemic control, among persons with type 2 diabetes mellitus.

Secondary objectives

To investigate the quality of sleep in individuals with T2DM with a standardized measure of sleep quality.

To examine how demographic factors (age, gender, marital status, and level of education) can affect the quality of sleep and glycemic control in individuals with type 2 diabetes mellitus.

To examine the clinical factors (duration of diabetes and type of treatment) to explore possible associations with sleep quality and glycemic control.

To find possible subgroups of patients who might be at risk of poor glycemic outcomes due to inadequate sleep.

## Materials and methods

Research design and methods

The study employed a cross-sectional research design to investigate the relationships between glycemic control and sleep quality in adults with type 2 diabetes mellitus (T2DM). A sample size of N = 385 was used to recruit the participants based on two outpatient healthcare centers within the city of Islamabad, Pakistan: Ahmed Medical Centre (n = 205) and Bilal Medical Centre (n = 180). The given methodology enabled a diverse range of individuals with varying socioeconomic and educational backgrounds, thereby maximizing the sample's representativeness. The structured questionnaires relied on demographics and two standardized components that addressed concerns regarding sleep quality and self-management of diabetes. The survey collected data on daily routines, sleeping habits, and diabetes self-care behaviors. These questionnaires were completed individually or with the assistance of trained research personnel, according to the participants' wishes and their level of understanding.

The methodology enabled the researchers to investigate the behavioral and life choices that could impact glycemic management, yielding findings that could be applied in community-based diabetes management.

Sample size and technique

The study was regarded as having an infinite population because the exact size of the population of individuals with type 2 diabetes in Pakistan who may experience altered sleep quality or glycemic control is not precisely known. In epidemiological research, when the total population size is very large or undefined, the assumption of an infinite population is commonly applied to simplify sample size calculation. This assumption ensures that the calculated sample size is both adequate and unbiased in relation to the population size estimates. However, it also means that the findings may be less generalizable to smaller, well-defined subgroups of patients, and the representativeness of the sample depends mainly on the sampling method rather than the population frame. The formula used to calculate the necessary sample size was as follows:

\[n = \frac{Z^2 \cdot p (1 - p)}{d^2}\]

In this formula, when Z is used, it denotes the standard score that corresponds to the required level of confidence, p is the presumed proportion, and d is the permissible margin of error. The level of confidence (95%) was standardly applied, and d was set at 0.05 (Z = 1.96). To provide the maximum sample size, the probability value of a false negative (p) was assumed to be 0.50 [13].

The study sample size of 385 participants was based on these parameters. Participants were sampled conveniently in community centers, as well as outpatient clinics and hospitals. Recruitment was conducted consecutively during routine outpatient visits, where all eligible individuals were invited to participate until the required sample size was achieved. This method facilitated the selection of individuals who were both eligible and willing to participate in the study throughout the data collection process.

Inclusion criteria

Participants recruited for the study were adults aged 18 years and above with a documented clinical diagnosis of type 2 diabetes mellitus (T2DM). The individual could be selected voluntarily and should be capable of giving informed consent and/or understanding, as well as filling out the questionnaire themselves or with minimal assistance. It ensured that the responses obtained were idealized and representative of what the participants were experiencing in terms of sleep quality and diabetes self-management.

Exclusion criteria

Those who had a known history of severe psychiatric illness or other conditions, such as cognitive impairment or neurological disorders, that could likely affect their ability to comprehend and fill in the questionnaire were excluded. Furthermore, persons with terminal illnesses, pregnant women, and those taking medications known to alter sleep significantly-wake schedules (e.g., sedative-hypnotics, benzodiazepines, or antidepressants) or directly influence glucose metabolism (e.g., systemic corticosteroids, certain antipsychotics, or beta-blockers) were also excluded, as these could bias the findings by either masking or exaggerating sleep disturbances or independently affecting glycemic control, thereby confounding the observed associations.

Data collection tools

In this research, a structured questionnaire was used, which had three major parts: demographic details, sleep quality assessment, and self-management practices assessment in the context of diabetes. The instruments contained standardized scales, along with researcher-created demographic questions, to facilitate relevance and complete coverage of the variables of interest. Both instruments were administered in a standardized manner, either self-completed or with the assistance of trained personnel to ensure comprehension. As they are self-report questionnaires, no calibration procedures were required.

Demographic information

The initial part involved collecting demographic data to explore potential relationships between participant characteristics and differences in sleep quality and glycemic control. The data gathered included age, gender, marital status, level of education, employment status, and lifestyle habits such as smoking. This part enabled descriptive profiling of the sample, allowing for the comparison of different subgroups.

Pittsburgh sleep quality index (PSQI)

In the present study, participants were assessed with the Pittsburgh Sleep Quality Index (PSQI) to evaluate their sleep quality. The PSQI consists of 19 items developed by Daniel J. Buysse and other researchers in 1989. It is a self-administered questionnaire that measures perceived sleep quality. These consist of seven components that are added to give a global score with a range of 0-21. Higher global scores indicate poorer sleep quality, with a score greater than five generally used as the cut-off point to differentiate between "good" and "poor" sleepers. Each item has a Likert scale of 0 to 3. The PSQI demonstrates satisfactory internal consistency, with a reported Cronbach's alpha value of 0.83. Since this is a freely accessible tool, no formal approval was necessary to use it in the study [14].

Diabetes self-management questionnaire

To measure self-care behaviors associated with diabetes management, the Diabetes Self-Management Questionnaire (DSMQ) developed by Schmitt et al. (2013) was used. This is a self-administered instrument designed to quantify individuals' ability to manage their diabetes in their daily lives. It is composed of 16 items that belong to four primary subscales: glucose management, dietary control, physical activity, and healthcare use. All items are measured using a 4-point Likert scale with a possibility of 0-3, where 0 ("that does not apply to me") and 3 ("applies to me to a great extent"). Subscale scores and total scores are obtained by summing the corresponding items. The higher the score, the better the diabetes self-management. The DSMQ scale exhibits good psychometric properties overall, with an alpha value of 0.84, indicating good internal consistency. The tool is also freely accessible online and can be used in both research and clinical settings [15].

Instrument permissions and licensing

Before data collection, formal permission was obtained for the use of both standardized tools. Permission to use the Pittsburgh Sleep Quality Index (PSQI) was granted by the University of Pittsburgh via email correspondence, specifically for this non-commercial academic study [13]. The approval outlined conditions for use in paper format and emphasized that separate permission would be required for future projects. For the Diabetes Self-Management Questionnaire (DSMQ), permission was obtained through the Mapi Research Trust under Special Terms Agreement No. 117885, granted to the principal investigator for academic use in this observational study [14]. The DSMQ was used in its original English version, and licensing details were aligned with the stated purpose as described in the agreement. Both tools were administered in accordance with the granted permissions and copyright guidelines.

Procedure

The study participants were recruited through an invitation, and they were expected to participate in the study after providing informed consent at selected outpatient healthcare facilities in Islamabad. The data collection procedure commenced in January 2025 and ended in May 2025, lasting a total of five months. Each participant was allowed to complete the questionnaire at their level of understanding, with or without the assistance of a trained research team member. To ensure confidentiality, the responses were not linked to personal identifiers, and all data were recorded anonymously. This methodology enabled the inclusion of adults from diverse socioeconomic and educational backgrounds, ensuring that all gathered information was representative, unbiased, and ethically processed.

Statistical analysis

The data analysis was performed using IBM Corp. Released 2020. IBM SPSS Statistics for Windows, Version 26. Armonk, NY: IBM Corp. The demographic information of the participants was summarized using descriptive statistics, including frequencies and percentages. The distribution of continuous variables, including the Pittsburgh Sleep Quality Index (PSQI) and the Diabetes Self-Management Questionnaire (DSMQ), was calculated using detrended Q-Q plots. Normality of the PSQI and DSMQ scores was assessed using Shapiro-Wilk tests, which indicated significant deviations from normality (p < 0.05). Therefore, non-parametric methods were applied for further analyses. An analysis of the correlation between PSQI and DSMQ scores was performed using Spearman's rank-order correlation. The Mann-Whitney U test was used to examine the differences in PSQI and DSMQ scores among the male and female participants. The Kruskal-Wallis H test was also used to determine the changes in sleep quality and diabetes self-management based on marital status. A simple regression was used to establish whether PSQI scores could predict DSMQ scores. Furthermore, chi-square tests have been used to determine the relationships between age, duration of diabetes, and the mode of diabetes treatment. Missing values were handled using pairwise deletion when nonessential items were missing at random, while records with substantial missing data were excluded from analysis. No multivariable adjustments for potential confounding factors were performed; instead, analyses focused on bivariate associations, which is acknowledged as a study limitation. The results of all statistical tests were analyzed with the level of significance (p < 0.05).

Ethical considerations

The study adhered to all the applicable ethical regulations concerning human research. Before collecting data, ethical approval was obtained from the Institutional Review Board (IRB) of Shifa International Hospital, Islamabad, IRB # 0307-25. This approval attests that the research adheres to the fundamental principles of ethics, including respect for individuals, protection of participant welfare, and confidentiality of individual information. The subjects were well-informed about the study's objectives, methods, potential risks, and expected benefits. All the participants signed written informed consent before being enrolled in the study. Participation in the survey was strictly voluntary, and participants were informed of their right to withdraw at any point without incurring any adverse effects. The responses were handled anonymously and were intended for use only in studies and academic work. Incomplete data were handled with much care to maintain the integrity of the data. When non-essential items were missing randomly from the participant's response data, the data were not disregarded; instead, they were analyzed using the pairwise deletion method to maximize the data. Significantly incomplete responses, however, were excluded to ensure the accuracy and reliability of the study findings in the final analysis.

## Results

Table 1 presents the demographic features of the respondents (N = 385). Most of the participants were between the ages of 31 and 40 years (N = 210, 54%), 41 and 50 years (N = 80, 21%), 51 years or above (N = 54, 14%), and 18 and 30 years (N = 41, 11%). The proportion of males (N = 195, 51%) was slightly higher than that of females (N = 190, 49%). The majority of participants were married (N = 179, 46%), while others were divorced (N = 105, 27%), widowed (N = 68, 18%), or single (N = 33, 9%). As far as education is concerned, a vast percentage of people had a graduate degree (N = 136, 35%); others were intermediate (N = 72, 19%), primary (N = 67, 17%), secondary/matric (N = 63, 16%), postgraduate (N = 25, 6%), and had no schooling (N = 22, 6%). Employment status revealed that the majority of them (N = 224, 58%) were unemployed, followed by students (N = 63, 16%), those who were employed (N = 55, 14%), and those who were retired (N = 43, 11%). Speaking of the duration of diabetes, the most frequent category was 1-5 years (N = 117, 30.4%), followed by 6-10 years (N = 99, 25.7%), more than 10 years (N = 95, 24.7%), and less than one year (N = 74, 19.2%). In managing diabetes, insulin injection (N = 116, 30%), physical exercise (N = 75, 19%), oral medication (N = 69, 18%), diet control (N = 68, 18%), or herbal/traditional remedies (N = 57, 15%) were used by the participants. The largest group (N = 132, 34%) monitored their blood sugar at home occasionally, while others did so regularly (N = 101, 26%) or not at all (N = 152, 40%). Hypertension (N = 123, 32%), cardiovascular disease (N = 96, 25%), thyroid disorder (N = 36, 9%), and kidney disease (N = 19, 5%) were found in the sample, and 111 (29%) of participants indicated no other chronic condition.

**Table 1:** Demographic Characteristics of Participants (N=385) f: frequency, %: percentage

Variable	f	%
Age	-	-
18-30 years	41	11
31-40 years	210	54
41-50 years	80	21
51 years or above	54	14
Gender	-	-
Male	195	51
Female	190	49
Marital status	-	-
Single	33	9
Married	179	46
Divorced	105	27
Widowed	68	18
Educational level	-	-
No formal education	22	6
Primary	67	17
Secondary/matric	63	16
Intermediate	72	19
Graduate	136	35
Postgraduate	25	6
Employment status	-	-
Student	63	16
Employed	55	14
Unemployed	224	58
Retired	43	11
Duration of type 2 diabetes	-	-
Less than 1 year	74	19.2
1-5 years	117	30.4
6-10 years	99	25.7
More than 10 years	95	24.7
Type of Diabetes Treatment	-	-
Oral medication	69	18
Insulin injections	116	30
Diet control	68	18
Physical exercise	75	19
Herbal/traditional remedies	57	15
Do you monitor your blood sugar levels at home?	-	-
Yes, regularly	101	26
Yes, occasionally	132	34
No	152	40
Do you have any other chronic health conditions?	-	-
None	111	29
Hypertension	123	32
Cardiovascular disease	96	25
Thyroid disorder	36	9
Kidney disease	19	5

**Figure 1:**
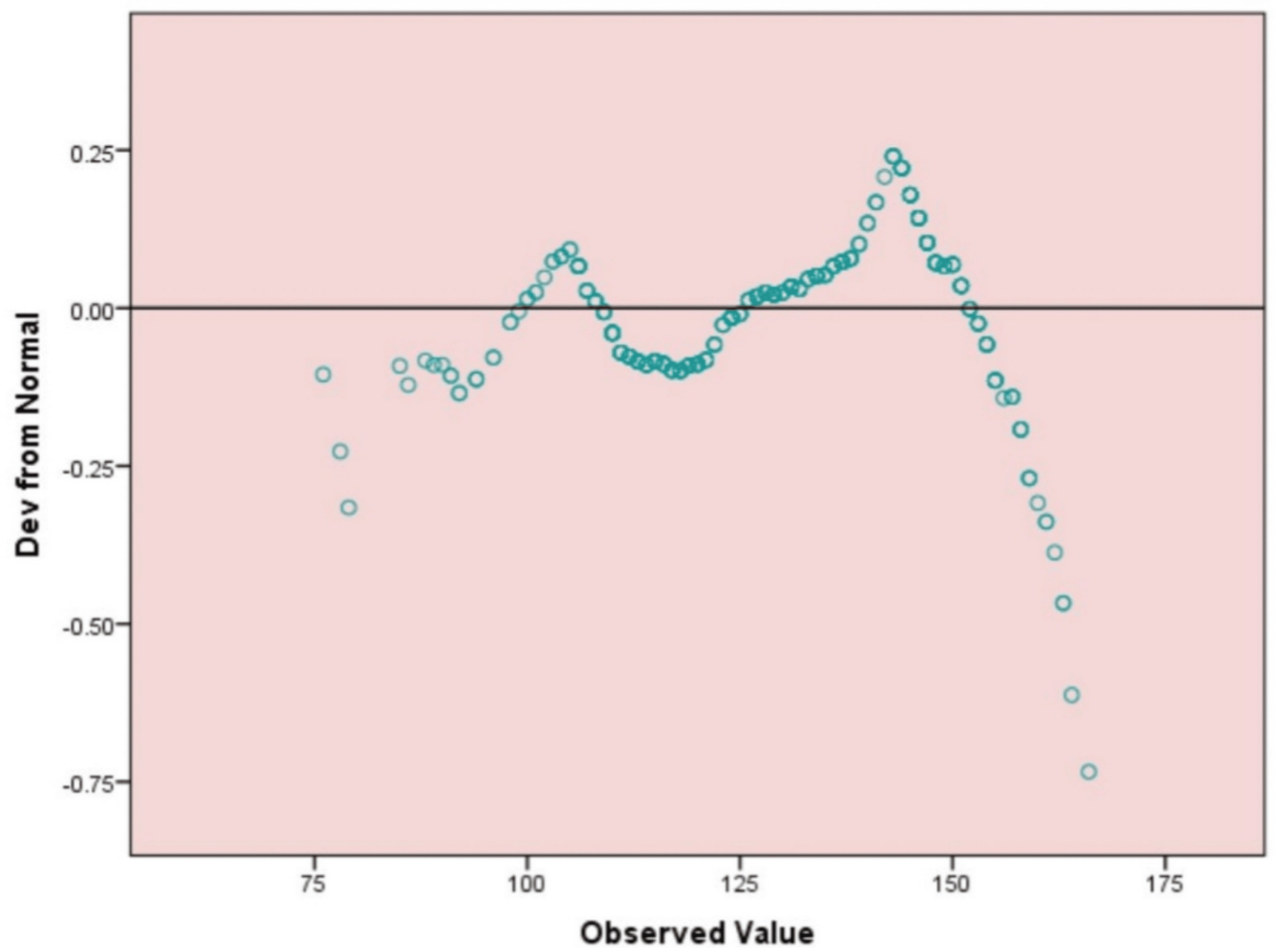
Detrended Q-Q Plot of Pittsburgh Sleep Quality Index Total Scores (PSQI) The image is created by the author. Detrended Q-Q plot of Pittsburgh Sleep Quality Index (PSQI) total scores showing notable deviations from normality, especially at the distribution tails.

Figure 1 shows the comparison of observed PSQI scores and expected values based on a normal distribution. The x-axis represents the observed values, and the y-axis represents the deviations from normality. The difference between the observed and the person's score is denoted as a dot in the graph. The wave-like distribution, particularly at both ends of the distribution (indicated by data points above and below the reference line), is evidence of significant deviations from normality. This suggests that the PSQI scores may not be normally distributed, and before applying a parametric form of statistics, we would need to transform the data.

**Figure 2:**
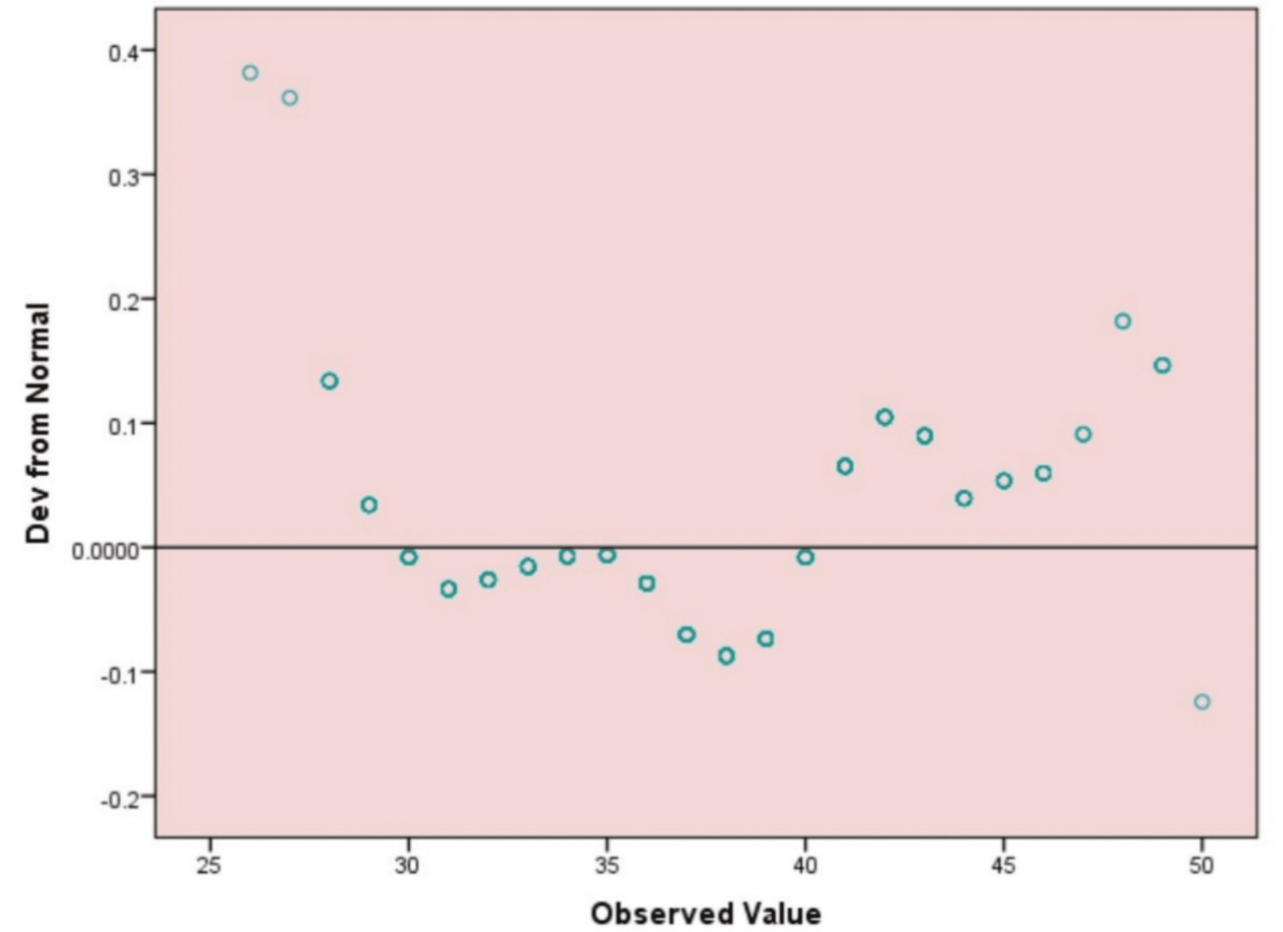
Detrended Q-Q Plot of Diabetes Self-Management Questionnaire Total Scores (DSMQ) The image is created by the author. Detrended Q-Q plot of Diabetes Self-Management Questionnaire (DSMQ) scores indicating mild deviations, suggesting a non-normal distribution pattern.

Figure 2 presents a detrended Q-Q plot of the Diabetes-Self-Management Questionnaire Total Scores (DSMQ). A majority of the data points lie near the horizontal line, indicating an approximation of normality; however, some outliers are evident. The deviations of the scores between 25-30 and approximately 50 indicate minimal and significant deviations, respectively, suggesting the possibility that the data may not follow a normal distribution. It means there is a possibility of additional analysis or transformation.

Table 2 shows the intercorrelation between PSQI and DSMQ. A statistically significant but weak negative correlation was observed (r = -0.119, p = 0.020), indicating that higher PSQI scores (poorer sleep quality) were associated with lower diabetes self-management scores among the study participants. Although statistically significant, the strength of the association was small, suggesting that while sleep quality and self-management are related to diabetes, additional factors are likely to play a more substantial role in influencing self-care behaviors.

**Table 2:** Intercorrelation Between the Study Variables r: correlation coefficient; *: p<0.05; correlation: spearman’s correlation

Variable	r	p
Pittsburgh Sleep Quality Index & Diabetes Self-Management Questionnaire	-0.119	0.020^*^

Table 3 shows the outcome of the Mann-Whitney U test comparing the scores of male (N = 195) and female (N = 190) participants on sleep quality and diabetes self-management. The female respondents scored higher (mean rank = 217.00) on the Pittsburgh Sleep Quality Index (PSQI) than the males (mean rank = 170.00), and this means that the female respondents exhibited poorer sleep quality as compared to the males with U = 13,500, Z = -2.81, and p = 0.005 (p < 0.01). Likewise, the Diabetes Self-Management Questionnaire (DSMQ) scores were also significantly higher in females (mean rank = 208.84) than in males (mean rank = 177.56), with U = 15,515, Z = -2.77, and p = 0.006 (*p < 0.01), indicating that females exhibit better self-management behaviors.

**Table 3:** Mann–Whitney U Test Comparing Male and Female Participants on Pittsburgh Sleep Quality Index and Diabetes Self-Management Questionnaire Scores N: number of participants, U: Mann–Whitney U statistic, Z: standardized test statistics, **: p<0.01 considered significant

Variable	Gender	N	Mean rank	U	Z	p
Pittsburgh Sleep Quality Index	Male	195	170.00	13,500	-2.81	0.005^**^
-	Female	190	217.00	-	-	-
Diabetes Self-Management Questionnaire	Male	195	177.56	15,515	-2.77	0.006^**^
-	Female	190	208.84	-	-	-

Table 4 represents the findings of the Kruskal-Wallis H test between the Pittsburgh Sleep Quality Index (PSQI) and Diabetes Self-Management Questionnaire (DSMQ) scores based on marital status groups: single (N = 33), married (N = 179), divorced (N = 105), and widowed (N = 68). The results indicated that there was a statistically significant difference between the marital status groups in PSQI scores, H (3) = 12.75, p = 0.005, and single participants had a general mean rank (240.00) and widowed participants a lower one (150.00), which implies that sleep quality was dependent on marital status. Likewise, there was a considerable variation in the DSMQ scores (H(3) = 15.30, p = 0.002), with widowed individuals showing the highest mean rank (240.00) and divorced individuals showing the lowest (180.00), indicating statistically significant variation in self-management behaviors by marital status.

**Table 4:** Kruskal–Wallis H Test Comparing Pittsburgh Sleep Quality Index and Diabetes Self-Management Questionnaire Scores by Marital Status N: number of participants, H(x2): Kruskal–Wallis test statistic, df: degree of freedom; **: p<0.01 considered significant

Variable	Marital status	N	Mean rank	H(x^2^)	df	p
Pittsburgh Sleep Quality Index	Single	33	240.00	-	^-^	-
-	Married	179	175.00	12.75	3	0.005^**^
-	Divorced	105	195.00	-	-	-
-	Widowed	68	150.00	-	-	-
Diabetes Self-Management Questionnaire	Single	33	230.00	-	^-^	-
-	Married	179	210.00	15.30	3	0.002^**^
-	Divorced	105	180.00	-	-	-
-	Widowed	68	240.00	-	-	-

Table 5 presents the findings of a linear regression model of the diabetes self-management scores (DSMQ) regressed against sleep quality (PSQI). The model reveals that PSQI made a significant contribution to the prediction of the DSMQ scores (B = -0.452, beta = -0.119, p = 0.021). Specifically, a one-unit increase in the PSQI score, which reflects poorer sleep quality, was associated with a 0.452-point reduction in the DSMQ score, indicating slightly poorer diabetes self-management. The confidence interval (95%) of the unstandardized coefficient was between the values of -0.84 and -0.07. The constant term was significant (B = 145.52, p < 0.001), indicating the mean DSMQ score at the zero point of the PSQI.

**Table 5:** Linear Regression Analysis Predicting Diabetes Self-Management Questionnaire (DSMQ) Scores using the Pittsburgh Sleep Quality Index (PSQI) B: coefficient, S.E: standard error, β: standardized coefficient, LL: lower limit, UL: upper limit; Cl: confidence interval, *: p<0.05, **: p<0.01 considered significant

Variable	B	95% Cl LL	UL	S.E	β	p-value
Constant	145.52	131.07	159.97	7.350	-	<0.001^**^
Pittsburgh Sleep Quality Index	-0.452	-0.84	-0.07	0.195	-0.119	0.021^*^

Table 6 examines the relationship between age categories and (a) the number of years of having type 2 diabetes mellitus and (b) the type of diabetes treatment. The correlation between age and duration of diabetes was also determined to be statistically significant using a chi-square test (χ² = 44.8, df = 9, p < 0.001); therefore, older age categories had a higher likelihood of experiencing diabetes over longer durations. Additionally, a strong correlation was observed between age and methods of diabetes management (χ² = 34.2, df = 1, p = 0.001), with younger subjects (18-30 years) engaging in more physical exercise and using herbal medications. In contrast, older subjects (51 years and above) were more likely to be taking insulin. These results indicate that there are age differences in the occurrence and treatment approaches of diabetes.

**Table 6:** Descriptive Statistics of Demographic Variables (Age, Duration of Type 2 Diabetes, Type of Diabetes Treatment) f: frequency; %: percentage; df: degree of freedom; x2: effect size; p: level of significance; p-values calculated using the chi-square test; the significance level is set at p < 0.05: *: p<0.05, **: p<0.01 considered significant

Variables	f	Less than 1 year	Duration of Type 2 Diabetes 1-5 years	6-10 years	More than 10 years	df	p	x^2^	Oral medication	Type of Diabetes Treatment Insulin injections	Diet control	Physical exercise	Herbal/traditional remedies	df	p	x^2^
Age	-	-	-	-		9	<0.001^**^	44.8	-	-	-	-	-	12	0.001^**^	34.2
18-30 years	41	6	23	9	3	-	-	-	7	8	5	14	7	-	-	-
31-40 years	210	46	62	62	40	-	-	-	38	70	45	38	19	-	-	-
41-50 years	80	16	18	22	24	-	-	-	19	15	14	13	19	-	-	-
51 years or above	54	6	14	6	28	-	-	-	5	23	4	10	12	-	-	-

## Discussion

This study reveals a statistically significant relationship between sleep quality and self-management in patients with type 2 diabetes mellitus (T2DM). Our study found a significant negative correlation between low sleep quality and diabetes self-management periods, implying that the worse the sleep, the less self-managed activity was performed. This aligns with prior research that found poor sleep and daytime interference to be related to higher levels of diabetes control issues and reduced adherence [16]. However, it is essential to note that the cross-sectional design of both studies, present and past, prevents conclusions about causality, whether inadequate sleep contributes to poor glycemic control or whether impaired glycemic control itself disrupts sleep patterns. This association could be a result of insufficient sleep and its effects on mood, energy, concentration, and motivation, which are core factors in achieving consistency in diabetes care practices.

The PSQI scores indicated that the quality of sleep of the female participants was significantly poorer compared to that of the male participants in our study. This finding is consistent with evidence from a previous study, which demonstrated that females were nearly twice as likely as males to report poor sleep quality, indicating a general sex-associated vulnerability present in the population [17]. However, our results showed an improvement in diabetes self-management among females, despite their lower quality of sleep. This concurs with the earlier work, which concluded that women had a higher proportion of increasing their diabetes knowledge, whereas men benefited from an increase in self-efficacy [18]. These disparities indicate that there is a possibility that gender-specific interventions are required to maximize diabetes education and self-care interventions.

In our research, individuals who were not married had significantly worse self-reported sleep quality than all the other marital groups, indicating that marital status and sleep are closely interrelated. This is confirmed through previous research, which has shown that unmarried adults have significantly poorer sleep quality, quantity, and timing [19]. Such disparities may be related to a lack of emotional support, higher stress, and abnormal lifestyle patterns characteristic of unmarried individuals, all of which can adversely affect sleep patterns. We have found that there is a substantial variation in diabetes self-management in different categories of marital status, whereby the widowed people showed the best self-management. Prior research also reported that self-management behaviors varied significantly according to marital status, which reinforced the participation of social and demographic determinants in influencing diabetes-related behaviors [20].

In our regression analysis, lower diabetes self-management was significantly predicted by poorer sleep quality. Several studies conducted previously have confirmed this finding: lower sleep quality results in more diabetes control issues and fewer self-health measures, highlighting the paramount significance of sleep in type 2 diabetes management [16].

We found that older participants also had significantly more prolonged type 2 diabetes, which makes the disease predominantly chronic among older adults. This coincides with the findings of the prior study, which demonstrated that those who had an earlier diagnosed age manifested greater risks of complications and death, further affirming the significance of early intervention and sustained disease management [21]. In our study, we found that older adults had a higher likelihood of using insulin, indicating the progression and development of the disease over time. A previous study had shown non-insulin-dependent diabetes to be more prevalent at diagnosis in older adults, which complemented our findings by suggesting that the use of insulin might increase with growing disease duration among older adults [22].

In general, this research strengthens the idea of mutual relations between sleep quality and glycemic control and the necessity to include sleep measurement in the process of diabetes management, especially in low- and medium-income countries such as Pakistan, where psychosocial factors, along with resource limitations, might increase the burden of sleep disturbances on diabetes treatment and management.

Limitations

Although the study offers valuable insights, several limitations should be noted. To begin with, the cross-sectional design of the study prevents the determination of causality between sleep quality and glycemic control. There is little evidence showing which of these two conditions, inappropriate sleep or inadequate self-management of diabetes, is the cause of the other condition. Secondly, self-report instruments (used in this study, e.g., the Pittsburgh Sleep Quality Index [PSQI] and the Diabetes Self-Management Questionnaire [DSMQ]) create the possibility of response bias and social desirability bias, which can interfere with the accuracy and validity of the self-reported data. Additionally, the researchers did not use an objective scale of sleep measurement, such as actigraphy or polysomnography, to confirm sleep patterns, which would have increased the study's validity. Another significant limitation is that the DSMQ measures self-management behaviors rather than direct biomarkers of glycemic control (e.g., HbA1c, fasting glucose, or CGM data). Therefore, our findings pertain to behavioral correlations of glycemic control, not clinical outcomes per se.

Although a convenience sampling approach seems an effective method for accessing participants within an outpatient healthcare facility, this type of sampling reduces the representativeness of the sample and introduces a risk of selection bias. Additionally, the sample was selected from among the healthcare units in Islamabad; therefore, the findings cannot be generalized to other regions or the rural population in Pakistan. Finally, the analysis did not account for statistical adjustments for potential confounders (such as comorbidities, lifestyle factors, or medication use), which may have influenced both sleep quality and diabetes self-management outcomes. Some unmeasured factors, such as psychological distress or undiagnosed sleep disorders (e.g., obstructive sleep apnea), could also have impacted the associations observed.

Future directions

To extend the results of this research, it would be desirable to conduct longitudinal studies that address the challenge of determining the time and causal connection between sleep quality and glycemic control in patients with type 2 diabetes. The inclusion of objective sleep measurements, such as actigraphy or sleep diaries, would provide an additional benefit in terms of accuracy and comprehensiveness of the information regarding the patient's sleep habits, offering a better insight into sleep trends. Interventional research is also necessary to determine whether improved sleep quality can lead to better glycemic outcomes through behavioral therapies, pharmacological methods, or sleep hygiene education. In addition, psychosocial variables, e.g., stress, depression, and anxiety, should be considered as possible mediators or moderators of the connection between sleep and diabetes in the future. The study should be extended to represent a more diverse range of geographic and financial contexts to increase the overall accuracy of the results. Finally, the inclusion of regular sleep testing in the protocol for diabetes management and education can present a new means of achieving a positive change in metabolic parameters on a long-term basis in a cost-effective manner.

## Conclusions

This research identified a statistically significant but weak negative association between sleep quality and diabetes self-management behaviors among patients with type 2 diabetes in the clinical setting in Pakistan. The insufficient self-care behaviors were also associated with poor sleep, making it critical to consider an additional measure of sleep in diabetes management. The results also suggest differences in sleep and glycemic control patterns based on gender and marital status, which strengthens the argument for the necessity of personal and setting-specific healthcare approaches. However, given the cross-sectional design, reliance on self-reported measures, and the behavioral (rather than clinical) nature of the outcomes, these results should be interpreted cautiously as associations rather than causal effects. Future studies incorporating objective sleep assessments, biomarkers of glycemic control (e.g., HbA1c), and longitudinal designs are needed to clarify directionality and strengthen the evidence base for sleep-focused interventions in diabetes care.
